# Recommended levels of calcium and non-phytate phosphorus for yellow-feathered broilers (finisher phase)

**DOI:** 10.5713/ab.21.0572

**Published:** 2022-04-29

**Authors:** Yibing Wang, Weiwei Wang, Sheng Zhang, Shouqun Jiang

**Affiliations:** 1 Institute of Animal Science, Guangdong Academy of Agricultural Sciences, State Key Laboratory of Livestock and Poultry Breeding, Key Laboratory of Animal Nutrition and Feed Science in South China, Ministry of Agriculture and Rural Affairs, Guangdong Provincial Key Laboratory of Animal Breeding and Nutrition, Guangzhou, 510640, China; 2 Institute of Grain Quality and Nutrition, Academy of National Food and Strategic Reserves Administration, Beijing, 100037, China

**Keywords:** Calcium and Phosphorus Metabolism, Meat Quality, Performance, Tibial Characteristics

## Abstract

**Objective:**

This study examined the effects of dietary calcium (Ca) and non-phytate phosphorus (NPP) on performance, tibial characteristics, meat quality and plasma biochemical variables in yellow-feathered broilers during 85 to 105 d of age.

**Methods:**

A total of 720 heads of 85-d broilers were allocated into 9 groups and provided with three levels of Ca (0.65%, 0.75%, 0.85%), and NPP (0.25%, 0.30%, 0.35%) in diets for 21 d.

**Results:**

The final body weight (FW), average daily gain (ADG), average daily feed intake (ADFI), and feed to gain ratio (F:G) were affected (p<0.05) by dietary Ca. From the quadratic regressions, the optimal level of Ca in diet were 0.71% for FW and ADG, and 0.67% for ADFI. Dietary Ca and NPP both significantly affected tibial breaking strength and density. From the quadratic regressions, the optimal level of Ca and NPP in diet were 0.81% and 0.37% for tibial density. The shear force of breast muscle of broilers given 0.75% or 0.85% Ca were lower than that in birds with 0.65% Ca and drip loss of birds given 0.65% or 0.75% Ca was lower than that in birds with 0.85% Ca (p<0.05). The drip loss of birds given 0.25% NPP was lowest among all NPP treatments (p<0.05). Calcium affected (p<0.05) the plasmal contents of phosphorus, osteocalcin (OC), parathyroid hormone (PTH) and calcitonin and the contents of OC and PTH were also influenced by dietary NPP.

**Conclusion:**

Dietary Ca and NPP level affected tibial characteristics, meat quality and biochemical variables in plasma of finisher-phase yellow-feathered broilers (85 to 105 d) and Ca also affected growth performance. Dietary 0.71% Ca and 0.30% NPP were enough for growth performance, while considering the growth performance, tibial characteristics, meat quality and biochemical variables together, 0.75% Ca and 0.37% NPP were recommended.

## INTRODUCTION

As the major mineral components of bone, calcium (Ca) and phosphorus (P) perform critical functions in skeletal integrity and a series of physiological processes in poultry [1–3]. Excessive Ca or P in diet, however, interferes with the availability of other minerals [4], implicates in decreased animal performance [5,6], and leads to environmental pollution [7]. Therefore, the appropriate provision of Ca and P diet is essential to broiler production.

Compared to commercial broilers, the growth rate and feed conversion rate of yellow-feathered broilers were slower. However, the meat of slow-growing broilers has a stronger flavor and better meat quality, which makes them the most preferred meat-type broilers in China, with almost the same annual sales as white-feathered broilers. Studies showed that flavor related substances in meat increased with age, such as inosinic acid and intramuscular fat [8]. In production, slow-growing yellow-feathered broilers would be raised for nearly four months to obtain better meat quality, and the recommended provision of Ca and non-phytate phosphorus (NPP) to yellow-feathered boilers at the last growth phase was still unknown. For commercial broilers at finisher phase, 0.80% Ca and 0.30% NPP were recommended by Nutrient Requirements of Poultry (National Research Council, 1994), therefore, a 3 (0.65%, 0.75%, 0.85% Ca)×3 (0.25%, 0.30%, 0.35% NPP) factorial arrangement was used in the current study. According to previous results, a hypothesis was formulated that for yellow-feathered broilers at the finisher phase (85 to 105 d), the provision of Ca and NPP influences the growth, tibial development, and meat quality. Moreover, in this study, the metabolism of Ca and P was also considered as important indexes, because that the serum Ca and P contents reflect the conditions of Ca and P homeostasis and necessary for normal physiological process and skeletal development [3,9].

## MATERIALS AND METHODS

### Ethics approval

All experimental procedures were approved by the Institutional Animal Care and Use Committee, Guangdong Academy of Agricultural Sciences (Guangzhou, China), with the approved number of GAASISA-2015-004.

### Experimental diets

Seven hundred and twelve yellow-feathered female broilers (85-d, a slow-growing meat-type breed) were used. The birds were previously provided with 0.90% Ca and 0.45% NPP during 1 to 28 d, 0.85% Ca and 0.40% NPP during 29 to 56 d, and 0.80% Ca and 0.35% NPP during 57 to 84 d. Three calculated dietary levels of Ca (0.65%, 0.75%, or 0.85%) and 3 calculated NPP levels (0.25%, 0.30%, or 0.35%) were used in a 3×3 factorial arrangement, each treatment with 4 replicates, and each replicate with 20 birds.

According to Chinese Feeding Standard of Chicken (2004), the experimental diets were formulated. Table 1 provides the details of composition and nutrient levels of the diets. The measurements of total P contents in the diet were performed by the colorimetric method of molybdo-vanadate and the Ca concentrations of the diet were determined by potassium permanganate method.

Broilers were provided with water and pelletized diets *ad libitum*. Daylight was replaced by incandescent bulbs providing 18-h lighting, and room temperature was kept at 26°C.

### Measurement of growth performance and meat quality

At the beginning (d 85) and end (d 105) of the experiment, Birds were weighed. The indicators of growth performance were measured as previously described [10].

At the end of the experiment (d 105), in each replicate, two birds (close to average final body weight [FW]) were chosen and deprived of feed overnight before slaughter. Breast muscles (the *pectoralis major*) were collected, and the drip loss and shear force were determined with the method of Wang et al [10].

### Measurement of tibial characteristics

Both sides of tibia were collected from every slaughtered bird and all adherent tissues were cleaned off. The diameter and length of right tibia were determined with a digital vernier calliper (500-151-30; Mitutoyo Corporation, Kawasaki, Japan), the weight was measured using analytical balance (ME204E; Mettler Toledo, Zurich, Switzerland), the bone mineral density was measured by an X-ray osteodensitometer (Lunar Prodigy, General Electric Company, Fairfield, CT, USA). For the left tibia, bone breaking strength was measured by a materials tester as described by Wang et al [11].

### Biochemical and endocrine concentrations

Colorimetric kits (H152, H207, and H153; Nanjing Jiancheng Bioengineering Institute, Nanjing, China) and a spectrophotometer (Biomate 5; Thermo, Rochester, NY, USA) were used to assay the contents of osteocalcin (OC), parathyroid hormone (PTH) and calcitonin (CT) in plasma. The plasmatic concentrations of P and Ca were assayed with colorimetric kits using an analyzer (CX5; Beckman Instruments, Inc., Brea, CA, USA).

### Statistical analysis

Multivariate analysis of variance (MANOVA) of SPSS 17.0 for Windows was used to examine the main effects (dietary Ca and NPP) and the interactive effect. When treatments effects were significant (p<0.05), the treatment means were further compared by Duncan multiple range tests. Polynomial regressions were fitted to test for effects (linearly and quadratically) in response to treatments. When a significant quadratic component was showed, Ca or NPP was estimated as the lower dietary level achieving 95% of the maximal of minimal response.

## RESULTS

### Performance

As provided in Table 2. Dietary Ca significantly influenced the FW, average daily gain (ADG), average daily feed intake (ADFI) and feed to gain ratio (F:G) (p<0.05). Broilers given 0.65% or 0.75% Ca had increased FW, ADG, ADFI and decreased (p<0.05) F:G than those received 0.85% Ca. Dietary NPP level had no influence on growth performance (p>0.05), and no interactions between Ca and NPP were observed on variables of performance of broilers from 85 to 105 d. From the quadratic regressions, the optimal level of Ca in diet were 0.71% for FW and ADG, and 0.67% for ADFI.

### Tibial characteristics

As presented (Table 3), the Ca and NPP level both influenced the breaking strength and density of tibia; there were no significant interactions. Breaking strength of birds given 0.85% Ca was higher than birds given 0.65% Ca (p<0.05), and the tibial density of broilers given 0.75% and 0.85% Ca was higher than birds fed 0.65%. Compared to those with 0.25% NPP, the breaking strength of broilers received 0.35% NPP increased (p<0.05), and compared to broilers given 0.25% NPP, tibial density of birds received 0.30% and 0.35% NPP was increased (p<0.05). From the quadratic regressions, the optimal level of Ca and NPP in diet were 0.81% or 0.37% for tibial density.

### Meat quality

Calcium and NPP levels in diet influenced drip loss and shear force of muscle (Table 4). The shear force of broilers that received 0.75% or 0.85% Ca decreased (p<0.05) compared to broilers on 0.65% Ca, and the drip loss of birds given 0.65% or 0.75% Ca decreased compared to broilers given 0.85% Ca (p<0.05). The shear force of muscle from broilers with 0.35% NPP was less than in birds given 0.30%, and the drip loss was reduced with 0.25% NPP compared to the other levels of NPP (p<0.05). On the above variables, there were no interactions between Ca and NPP. From the quadratic regressions, the optimal level of Ca in diet were 0.80% and 0.60% for shear force and drip loss, respectively.

### Plasma Ca, P, and endocrine measurements

The Ca level in diet influenced (p<0.05) the content of P, OC, PTH, and CT, as presented in Table 5. In broilers provided with 0.85% Ca, the OC content increased (p<0.05) compared to broilers given less Ca, PTH content was higher than with 0.75% Ca, and plasma P content exceeded that in birds fed 0.65% Ca. The content of CT in birds given 0.65% Ca was higher than in birds given 0.75% Ca. The dietary NPP levels also affected contents of OC and PTH (p<0.05). Concentrations of OC increased in birds given 0.25% and 0.30% NPP over those with 0.35% NPP, and the PTH content increased in broilers provided with 0.35% NPP over those given 0.35% NPP; there was an interaction between Ca and NPP in influencing plasma PTH. Neither dietary Ca nor NPP affected the content of Ca in plasma (p>0.05).

## DISCUSSION

As the most abundant minerals in the body, provision of appropriate amounts of dietary Ca and P is critical for poultry production. In addition to their accretion in bone, they are essential for biological processes such as intracellular signaling, and muscle contractions [1,10]. Excessive dietary Ca and P, however, have a harmful impact on growth of broilers [6,12,13]. The current finding showed that the FW, ADG, ADFI, and F:G were influence by Ca level in diet with birds fed 0.85% Ca having lower FW, ADG, ADFI, and higher F:G than those fed 0.65% or 0.75% Ca. Similarly, Hamdi et al [6] showed that higher Ca levels was deleterious for feed intake and body weight of Ross chicks, and to achieve better performance, a lower dietary level of Ca was preferable. Gautier et al [12] found that reduction of dietary Ca improved feed conversion rate of broilers compared with birds received high Ca levels. Excessive dietary Ca complexes with other molecules and interfered with the availability of other minerals [12,14,15]. Shafey et al [13] showed that a high level of dietary Ca decreased the ratio of zinc associated with small complexes and soluble magnesium in meat chickens. Calcium reacted with free saturated fatty acid forming insoluble soaps, thus reducing the dietary energy digestibility [6,16]. The above indicated that Ca level in diets should be optimized, based on need, and there also should be more in-depth and careful study for different animal strains. Dietary NPP level was found to not influence performance of broilers studied here. Considering variables of growth performance, the optimal level of dietary Ca and NPP for yellow-feathered broilers from 85 to 105 d were 0.71% and 0.25%, respectively.

Calcium and phosphorus, the major mineral components of bone [17], are fundamental for growth and bone development and play critical roles on rigidity and compressive strength of bone [18]. The present research found that Ca and NPP level both significantly affected the breaking strength and density of tibias of yellow-feathered broilers. Typically, density, breaking strength, mineral, and ash content of bone are used as indicators of bone health [19,20]. In the past, sufficient research has proved that Ca and NPP are beneficial for the bone status and skeletal health and increasing their levels would lead to higher tibial weight [6], a greater ash content of tibia [1] and increased femur breaking strength and stiffness [21] of broilers. Gautier et al [12] suggested density of bone may be a more precise way because it measures the mass of material per unit volume of bone in the matrix. The first increasing and then decreasing effect of Ca or NPP on tibial breaking strength and density indicated that bone damage would be induced with excessive Ca and NPP in diet, which was similar earlier findings [5,22]. That might be explained that excessive Ca in diet reduced the availability of minerals, including P, from Ca chelating molecules in the digesta and reducing their availability for absorption [12,14]. From the quadratic regressions for tibial density, the optimal level of Ca and NPP in diet were the in yellow-feathered broilers aged 85 to 105 d were 0.81% and 0.37%, respectively. Interestingly, those levels exceed what was needed for growth performance, consistent with a previous study using Ross 308 broilers [12].

The need has been underlined worldwide for improved meat quality [23], which was indirectly assessed here using objective indices including pH, meat color, shear force and drip loss [24]. Shear force of breast muscle is one of the main sensorial attributes related to acceptability of customers, indicating meat tenderness [25] and drip loss indicates water holding capacity. In the current research on yellow-feathered broilers, dietary Ca and NPP level affected drip loss and shear force of breast muscle. Insufficient or excessive dietary Ca adversely affected these two indicators, and for meat quality, the optimal level of dietary Ca was 0.75%. There has been little research on possible influence of Ca and NPP levels on meat quality. Like the current finding of NPP influencing shear force of breast muscle, Li et al [26] had found this in broilers. Also, our previous study on broilers aged 56 to 84 d also showed that dietary Ca level significantly affected shear force and dietary NPP level affected drip loss of breast muscle. The change of meat quality in the current research might be related to oxidative stress because poor meat quality of broilers is commonly seen along with oxidative damage, and NPP could reduce lipid and protein oxidation [10]. Under the present experimental conditions, for yellow-feathered broilers at finisher phase, dietary level of 0.75% Ca and 0.25% NPP achieved the best meat quality.

The serum Ca and P contents reflect the conditions of Ca and P homeostasis, which in a normal range are necessary for normal physiological process and skeletal development, and the deficiency of one of them interferes with homeostasis and absorption of the other [3]. For example, low P concentration in plasma leads to the activation of osteoclasts that, in turn, leads to increased bone resorption for maintaining a normal serum P level and simultaneously increasing serum Ca level [3]. The current research found that Ca in diet affected the content of P in plasma and in birds fed 0.85% Ca, plasma P content exceeded that in birds fed 0.65% Ca. Similarly, Rama Rao et al [27] suggested that, receiving suboptimal dietary Ca and NPP level in Cobb 100 broilers decreased Ca and inorganic P in serum. Li et al [3] found that dietary Ca deficiency did harm to bone formation by increasing Ca concentration and decreasing serum P content in serum of Arbor Acres male broilers, which may be a due to reduced available P for absorption in the digestive tract by forming a flocculent precipitate of Ca phosphate when the proportions of dietary Ca and NPP is disproportionate. Calcium and P metabolic utilization and homeostasis in plasma are regulated by a series of endocrine factors. In the present study, dietary Ca level influenced the content of OC, PTH and CT, dietary NNP levels affected OC and PTH, in plasma of yellow-feathered broilers. Parathyroid hormone is a regulator which acts importantly in Ca and P contents in the bone and blood [28]. Calcitonin is kind of an inhibitor of bone resorption, resulting in a reduce Ca content in plasma [29]. Osteocalcin regulates bone mineralization and bone turnover, and the OC levels in serum are used to evaluate bone metabolism, as an indicator of bone formation [30]. As Gautier et al [12] suggested, when Ca is deficient, PTH is produced by the parathyroid gland thus restrains Ca through promoting renal Ca re-absorption and stimulating released renal P, and when Ca is excessive, the CT is produced by the parathyroid gland to inhibit renal Ca re-absorption and thus increases Ca excretion via the urine. In the study of Manangi et al [29], low P in plasma increases ionized Ca which in turn suppresses the secretion of the PTH and exceed NPP level increased gene expression and secretion of PTH and led to a continuous process of bone resorption [28].

## CONCLUSION

Dietary Ca and NPP affected Ca and P metabolism in plasma, and further Ca and P deposition in bones, thus resulting in influenced growth performance, tibial characteristics, meat quality of finisher-phase yellow-feathered broilers (85 to 105 d). Different Ca and NPP levels should be considered to meet different performance, and taking all variables together, 0.75% Ca and 0.37% NPP were recommended for broilers at finisher phase.

## Figures and Tables

**Table 1:** Composition and nutrient contents of diets (85 to 105 d)

Calcium NPP	0.65%			0.75%			0.85%		
		
	0.25%	0.30%	0.35%	0.25%	0.30%	0.35%	0.25%	0.30%	0.35%
Ingredients (%)									
Corn	81.30	81.30	81.30	81.30	81.30	81.30	81.30	81.30	81.30
Soybean meal	11.00	11.00	11.00	11.00	11.00	11.00	11.00	11.00	11.00
Corn gluten meal	1.00	1.00	1.00	1.00	1.00	1.00	1.00	1.00	1.00
Limestone	1.02	0.83	0.64	1.30	1.11	0.92	1.58	1.39	1.20
Calcium monohydrogen phosphate	0.91	1.20	1.49	0.91	1.20	1.49	0.91	1.20	1.49
NaCl	0.32	0.32	0.32	0.32	0.32	0.32	0.32	0.32	0.32
DL-Met (99%)	0.20	0.20	0.20	0.20	0.20	0.20	0.20	0.20	0.20
L-LysHCL (78%)	0.38	0.38	0.38	0.38	0.38	0.38	0.38	0.38	0.38
Vitamin and mineral premix^1)^	1.00	1.00	1.00	1.00	1.00	1.00	1.00	1.00	1.00
Zeolite powder	2.87	2.77	2.67	2.59	2.49	2.39	2.31	2.21	2.11
Total	100.00	100.00	100.00	100.00	100.00	100.00	100.00	100.00	100.00
Nutrient contents^2)^									
Metabolizable energy (MJ/kg)	12.30	12.30	12.30	12.30	12.30	12.30	12.30	12.30	12.30
Crude protein (%)	13.00	13.00	13.00	13.00	13.00	13.00	13.00	13.00	13.00
Calcium (%)	0.65	0.65	0.65	0.75	0.75	0.75	0.85	0.85	0.85
Non-phytate phosphorus (%)	0.25	0.30	0.35	0.25	0.30	0.35	0.25	0.30	0.35
Lysine (%)	0.81	0.81	0.81	0.81	0.81	0.81	0.81	0.81	0.81
Methionine (%)	0.41	0.41	0.41	0.41	0.41	0.41	0.41	0.41	0.41
Methionine+cystine (%)	0.62	0.62	0.62	0.62	0.62	0.62	0.62	0.62	0.62
Calcium (%, analyzed)	0.66	0.66	0.64	0.75	0.73	0.76	0.87	0.86	0.86
Phosphorus (%, analyzed)	0.46	0.50	0.56	0.47	0.51	0.55	0.46	0.53	0.56

1) Premix provided the following per kilogram of diets during 85 to 105 days of age: Vit D_3_ 1,000 IU, Vit E 20 IU, Vit K_3_ 4 mg, Vit B_1_ 1.8 mg, Vit B_2_ 8 mg, Vit B_6_ 3.5 mg, Vit B_12_ 0.01 mg, chloride 500 mg, niacin 44 mg, pantothenic acid 10 mg, folic acid 0.55 mg, biotin 0.15 mg, Fe 80 mg, Cu 8 mg, Mn 80 mg, Zn 60 mg, I 0.35 mg, Se 0.15 mg.

2) Except where indicated, nutrient levels were all calculated value.

**Table 2:** Growth performance of broilers from 85 to 105 days of age with different calcium (Ca) and non-phytate phosphorus

Treatment		Body weight (d 85, g)	Body weight (d 105, g)	ADG (g)	ADFI (g)	F:G

Ca (%)	NPP (%)					
0.65	0.25	1,164.37	1,392.76	11.42	77.71	7.08
	0.30	1,169.37	1,372.27	10.14	72.81	7.41
	0.35	1,170.62	1,389.53	10.94	79.51	7.48
0.75	0.25	1,168.75	1,377.83	10.45	74.75	7.30
	0.30	1,168.75	1,395.06	11.31	77.64	7.58
	0.35	1,164.37	1,406.01	12.08	73.45	6.30
0.85	0.25	1,170.62	1,374.53	10.19	75.04	7.69
	0.30	1,170.00	1,324.05	7.70	66.10	8.80
	0.35	1,168.75	1,315.43	7.33	65.44	9.40
SEM		1.61	27.32	1.38	3.40	0.90
Main effect means						
0.65		1,168.12	1,384.85^a^	10.83^a^	76.67^a^	7.32^b^
0.75		1,167.29	1,392.97^a^	11.28^a^	75.28^a^	7.06^b^
0.85		1,169.79	1,338.00^b^	8.41^b^	68.86^b^	8.63^a^
SEM		0.90	15.75	0.80	1.99	0.52
	0.25	1,167.91	1,381.71	10.69	75.83	7.36
	0.30	1,169.37	1,363.79	9.72	72.18	7.93
	0.35	1,167.91	1,370.33	10.12	72.80	7.73
	SEM	0.90	15.75	0.80	1.99	0.52
p-values						
Ca		0.271	0.024	0.019	0.007	0.046
Linear			0.034	0.029	0.003	0.073
Quadratic			0.024	0.019	0.007	0.063
NPP		0.295	0.696	0.660	0.245	0.728
Ca×NPP		0.196	0.527	0.518	0.096	0.644

NPP, non-phytate phosphorus; ADG, average daily gain; ADFI, average daily feed intake; F:G, feed to gain ratio; SEM, standard error of the mean.

Quatratic regression equations based on the Ca level (%): Body weight_d 105_ (g) = −3,154.10(Ca)^2^+4,496.91(Ca)−205.53, R^2^ = 0.212, p = 0.024, which yielded the optimized dietary Ca level of 0.71%; ADG (g) = −166.04(Ca)^2^+236.93(Ca)−73.02, R^2^ = 0.215, p = 0.019, which yielded the optimized dietary Ca level of 0.71%; ADFI (g) = −251.63(Ca)^2^+338.36(Ca)−36.94, R^2^ = 0.258, p = 0.007, which yielded the optimized dietary Ca level value of 0.67%.

a,b Within a column, means with different lowercase superscripts was significant different (p<0.05).

**Table 3:** Tibial characteristics of broilers at 105 days of age with different calcium (Ca) and non-phytate phosphorus

Treatment		Weight (g)	Length (cm)	Diameter (mm)	Breaking strength (kgf)	Density (g/cm^2^)

Ca (%)	NPP (%)					
0.65	0.25	7.80	103.55	4.78	20.59	0.147
	0.30	8.33	105.04	4.67	20.92	0.154
	0.35	8.54	106.79	4.83	23.27	0.164
0.75	0.25	8.15	104.63	4.77	21.06	0.144
	0.30	8.68	106.06	4.67	23.01	0.216
	0.35	7.87	101.95	4.70	24.65	0.211
0.85	0.25	7.94	105.61	4.70	23.17	0.187
	0.30	8.36	105.02	4.77	24.50	0.181
	0.35	8.06	106.17	4.58	26.32	0.211
SEM		0.22	1.15	0.07	1.30	0.013
Main effect means						
0.65		8.22	105.13	4.76	21.59^b^	0.155b
0.75		8.23	104.22	4.71	22.91^ab^	0.190a
0.85		8.12	105.60	4.68	24.66^a^	0.193a
SEM		0.13	0.66	0.04	0.75	0.008
	0.25	7.96	104.60	4.75	21.60^b^	0.159b
	0.30	8.45	105.38	4.70	22.81^ab^	0.183a
	0.35	8.16	104.97	4.71	24.75^a^	0.195a
	SEM	0.13	0.66	0.04	0.75	0.013
p-values						
Ca		0.819	0.421	0.495	0.036	<0.001
Linear					0.010	0.002
Quadratic					0.055	0.002
NPP		0.062	0.764	0.710	0.028	0.002
Linear					0.008	0.004
Quadratic					0.060	0.012
Ca×NPP		0.568	0.245	0.106	0.305	0.986

NPP, non-phytate phosphorus; SEM, standard error of the mean.

Quatratic regression equations based on the Ca level (%): Density (g/cm^2^) = −1.63(Ca)^2^+2.63(Ca)−0.87, R^2^ = 0.210, p = 0.002, which yielded the optimized dietary Ca level of 0.81%; quatratic regression equations based on the P level (%): density (g/cm^2^) = −2.43(P)^2^+1.82(P)−0.14, R^2^ = 0.210, p = 0.002, which yielded the optimized dietary P level of 0.37%.

a,b Within a column, means with different lowercase superscripts was significant different (p<0.05).

**Table 4:** Indicators of meat quality of broilers at 105 days of age with different calcium (Ca) and non-phytate phosphorus (NPP)

Treatment		Shear force (kgf)	Drip loss (%)

Ca (%)	NPP (%)		
0.65	0.25	4.70	1.25
	0.30	4.94	1.43
	0.35	4.77	1.62
0.75	0.25	3.77	1.46
	0.30	3.21	1.81
	0.35	2.83	1.52
0.85	0.25	2.87	1.77
	0.30	4.13	2.10
	0.35	2.75	1.94
SEM		0.40	0.14
Main effect means			
0.65		4.80^a^	1.43^b^
0.75		3.27^b^	1.60^b^
0.85		3.25^b^	1.93^a^
SEM		0.23	0.07
	0.25	3.78	1.49^b^
	0.30	4.09	1.78^a^
	0.35	3.45	1.69^a^
	SEM	0.23	0.07
p-values			
Ca		<0.001	<0.001
Linear		<0.001	<0.001
Quadratic		0.003	<0.001
NPP		0.068	0.002
Linear		0.388	0.072
Quadratic		0.253	0.059
Ca×NPP		0.070	0.163

NPP, non-phytate phosphorus; SEM, standard error of the mean.

Quatratic regression equations based on the Ca level (%): Shear force (kgf) = 75.50(Ca)^2^−121.02(Ca)−51.57, R^2^ = 0.421, p = 0.003, which yielded the optimized dietary Ca level of 0.80%; Drip loss (%) = 8.50(Ca)^2^−10.26(Ca)+4.51, R^2^ = 0.389, p<0.001, which yielded the optimized dietary Ca level of 0.60%.

a,b Within a column, means with different lowercase superscripts was significant different (p<0.05).

**Table 5:** Plasma biochemical variables of broilers at 105 days of age with different calcium (Ca) and non-phytate phosphorus

Treatment		P (mmol/L)	Ca (mmol/L)	OC (ng/mL)	PTH (pg/mL)	CT (pg/mL)

Ca (%)	NPP (%)					
0.65	0.25	1.35	2.51	3.59^b^	16.48	50.99^a^
	0.30	1.29	3.27	3.08^b^	15.09	49.03^ab^
	0.35	1.35	2.68	3.15^b^	16.92	45.77^abc^
0.75	0.25	1.42	3.45	3.98^b^	12.54	37.45^d^
	0.30	1.35	3.10	3.43^b^	9.54	46.33^abc^
	0.35	1.45	2.91	3.23^b^	14.83	46.02^abc^
0.85	0.25	1.42	2.91	5.25^a^	17.65	44.56^bc^
	0.30	1.50	2.81	4.92^a^	15.17	47.83^ab^
	0.35	1.58	3.13	2.73^b^	20.57	40.60^c^
SEM		0.07	0.24	0.40	1.90	2.20
Main effect means						
0.65		1.33^b^	2.79	3.29^b^	16.16^a^	48.76^a^
0.75		1.41^ab^	3.17	3.52^b^	12.31^b^	43.10^b^
0.85		1.50^a^	2.95	4.24^a^	17.79^a^	44.33^ab^
SEM		0.04	0.14	0.23	1.10	1.27
	0.25	1.39	2.96	4.23^a^	15.51^ab^	44.33
	0.30	1.38	3.04	3.85^a^	13.26^b^	47.73
	0.35	1.46	2.91	3.03^b^	17.44^a^	43.91
	SEM	0.04	0.14	0.25	1.10	1.27
p-values						
Ca		0.008	0.537	0.002	0.001	0.008
Linear		0.002		0.014	0.129	0.049
Quadratic		0.006		0.028	0.001	0.020
NPP		0.504	0.344	0.001	0.023	0.126
Linear				0.002	0.143	
Quadratic				0.006	0.039	
Ca×NPP		0.297	0.295	0.007	0.141	0.008

NPP, non-phytate phosphorus; P, phosphorus; OC, osteocalcin; PTH, parathyroid hormone; CT, calcitonin; SEM, standard error of the mean.

P (mmol/L) = 2.73(Ca)^2^−3.14(Ca)+2.19, R^2^ = 0.2203, p = 0.006, which yielded the optimized dietary Ca level of 0.58%; OC (ng/mL) = 36.80(Ca)^2^−50.43(Ca) +20.52, R^2^ = 0.147, p = 0.028, which yielded the optimized dietary Ca level of 0.69%; PTH (pg/mL) = 448.06 (Ca)^2^−660.17(Ca)+254.46, R^2^ = 0.252, p = 0.001, which yielded the optimized dietary Ca level of 0.74%; CT (pg/mL) = 367.32 (Ca)^2^−572.38(Ca)+265.13, R^2^ = 0.160, p = 0.020, which yielded the optimized dietary Ca level of 0.78%; Quatratic regression equations based on the P level (%): OC (ng/mL) = −88.58(P)^2^+41.12(P)−0.51, R^2^ = 0.196, p = 0.006, which yielded the optimized dietary P level of 0.23%; CT (pg/mL) =1,119.09(P)^2^−648.53(P)−106.46, R^2^ = 0.129, p = 0.039, which yielded the optimized dietary P level of 0.29%.

a–c Within a column, means with different superscripts differ significantly (p<0.05).

## References

[1] Majeed S, Qudsieh R, Edens FW, Brake J (2020). Limestone particle size, calcium and phosphorus levels, and phytase effects on live performance and nutrients digestibility of broilers. Poult Sci.

[2] Suttle NF (2010). Mineral nutrition of livestock.

[3] Li T, Xing G, Shao Y (2020). Dietary calcium or phosphorus deficiency impairs the bone development by regulating related calcium or phosphorus metabolic utilization parameters of broilers. Poult Sci.

[4] Driver JP, Pesti GM, Bakalli RI, Edwards HJ (2005). Calcium requirements of the modern broiler chicken as influenced by dietary protein and age. Poult Sci.

[5] Calvo MS, Tucker KL (2013). Is phosphorus intake that exceeds dietary requirements a risk factor in bone health?. Ann NY Acad Sci.

[6] Hamdi M, López-Vergé S, Manzanilla EG, Barroeta AC, Pérez JF (2015). Effect of different levels of calcium and phosphorus and their interaction on the performance of young broilers. Poult Sci.

[7] Mitchell RD, Edwards HJ (1996). Effects of phytase and 1,25-dihydroxycholecalciferol on phytate utilization and the quantitative requirement for calcium and phosphorus in young broiler chickens. Poult Sci.

[8] Cui XY, Gou ZY, Jiang SQ, Jiang ZY (2019). Research advance of formation mechanism of chicken meat flavor and regulation. China J Anim Nutr.

[9] Proszkowiec-Weglarz M, Angel R (2013). Calcium and phosphorus metabolism in broilers: Effect of homeostatic mechanism on calcium and phosphorus digestibility. J Appl Poult Res.

[10] Wang YB, Wang WW, Li L, Gou ZY, Lin XJ, Jiang SQ (2021). Effects and interaction of dietary calcium and nonphytate phosphorus for slow-growing yellow-feathered broilers between 56 and 84 d of age. Poult Sci.

[11] Wang S, Chen W, Zhang HX, Ruan D, Lin YC (2014). Influence of particle size and calcium source on production performance, egg quality, and bone parameters in laying ducks. Poult Sci.

[12] Gautier AE, Walk CL, Dilger RN (2017). Influence of dietary calcium concentrations and the calcium-to-non-phytate phosphorus ratio on growth performance, bone characteristics, and digestibility in broilers. Poult Sci.

[13] Shafey TM, McDonald MW, Dingle JG (1991). Effects of dietary calcium and available phosphorus concentration on digesta pH and on the availability of calcium, iron, magnesium and zinc from the intestinal contents of meat chickens. Br Poult Sci.

[14] Qian H, Kornegay ET, Denbow DM (1997). Utilization of phytate phosphorus and calcium as influenced by microbial phytase, cholecalciferol, and the calcium: total phosphorus ratio in broiler diets. Poult Sci.

[15] Sebastian SS, Touchburn P, Chavez ER, Lague PC (1996). Efficacy of supplemental microbial phytase at different dietary calcium levels on growth performance and mineral utilization on broilers chickens. Poult Sci.

[16] Gowen JW, Tobey ER (1931). On the mechanism of milk secretion: the influence of insulin and phloridzin. J Gen Physiol.

[17] Proszkowiec-Weglarz M, Schreier LL, Miska KB, Angel R, Kahl S, Russell B (2019). Effect of early neonatal development and delayed feeding post-hatch on jejunal and ileal calcium and phosphorus transporter genes expression in broiler chickens. Poult Sci.

[18] Rath NC, Huff GR, Huff WE, Balog JM (2000). Factors regulating bone maturity and strength in poultry. Poult Sci.

[19] Bradbury EJ, Wilkinson SJ, Cronin GM, Thomson PC, Bedford MR, Cowieson AJ (2014). Nutritional geometry of calcium and phosphorus nutrition in broiler chicks. Growth performance, skeletal health and intake arrays. Animal.

[20] Shim MY, Karnuah AB, Mitchell AD, Anthony NB, Pesti GM, Aggrey SE (2012). The effects of growth rate on leg morphology and tibia breaking strength, mineral density, mineral content, and bone ash in broilers. Poult Sci.

[21] Swiatkiewicz S, Koreleski J, Arczewska-Wloek A (2011). Effect of inulin and oligofructose on performance and bone characteristics of broiler chickens fed on diets with different concentrations of calcium and phosphorus. Br Poult Sci.

[22] Powell S, Bidner TD, Southern LL (2011). Phytase supplementation improved growth performance and bone characteristics in broilers fed varying levels of dietary calcium. Poult Sci.

[23] Maiorano G, Sobolewska A, Cianciullo D (2012). Influence of in ovo prebiotic and synbiotic administration on meat quality of broiler chickens. Poult Sci.

[24] Joo ST, Kim GD, Hwang YH, Ryu YC (2013). Control of fresh meat quality through manipulation of muscle fiber characteristics. Meat Sci.

[25] Chen KL, Chen TT, Lin KJ, Chiou PWS (2007). The effects of caponization age on muscle characteristics in male chicken. Asian-Australas J Anim Sci.

[26] Li XK, Wang JZ, Wang CQ (2016). Effect of dietary phosphorus levels on meat quality and lipid metabolism in broiler chickens. Food Chem.

[27] Rama Rao SV, Raju MVLN, Panda AK, Sunder GS, Sharma RP (2006). Effect of high concentrations of cholecalciferol on growth, bone mineralization, and mineral retention in broiler chicks fed suboptimal concentrations of calcium and nonphytate phosphorus. J Appl Poult Res.

[28] Nari N, Ghasemi HA (2020). Growth performance, nutrient digestibility, bone mineralization, and hormone profile in broilers fed with phosphorus-deficient diets supplemented with butyric acid and Saccharomyces boulardii. Poult Sci.

[29] Manangi MK, Maharjan P, Coon CN (2018). Effect of different concentrations of dietary P and Ca on plasma inorganic P and urinary P excretion using noncolostomized and colostomized broilers. Poult Sci.

[30] Neve A, Corrado A, Cantatore FP (2013). Osteocalcin: skeletal and extra-skeletal effects. J Cell Physiol.

